# Supplementary figures and images for: New viral biogeochemical roles revealed through metagenomic analysis of Lake Baikal
Source: Microbiome. 2020 Nov 19;8:163. doi: 10.1186/s40168-020-00936-4 (PMC7678222; doi:10.1186/s40168-020-00936-4)

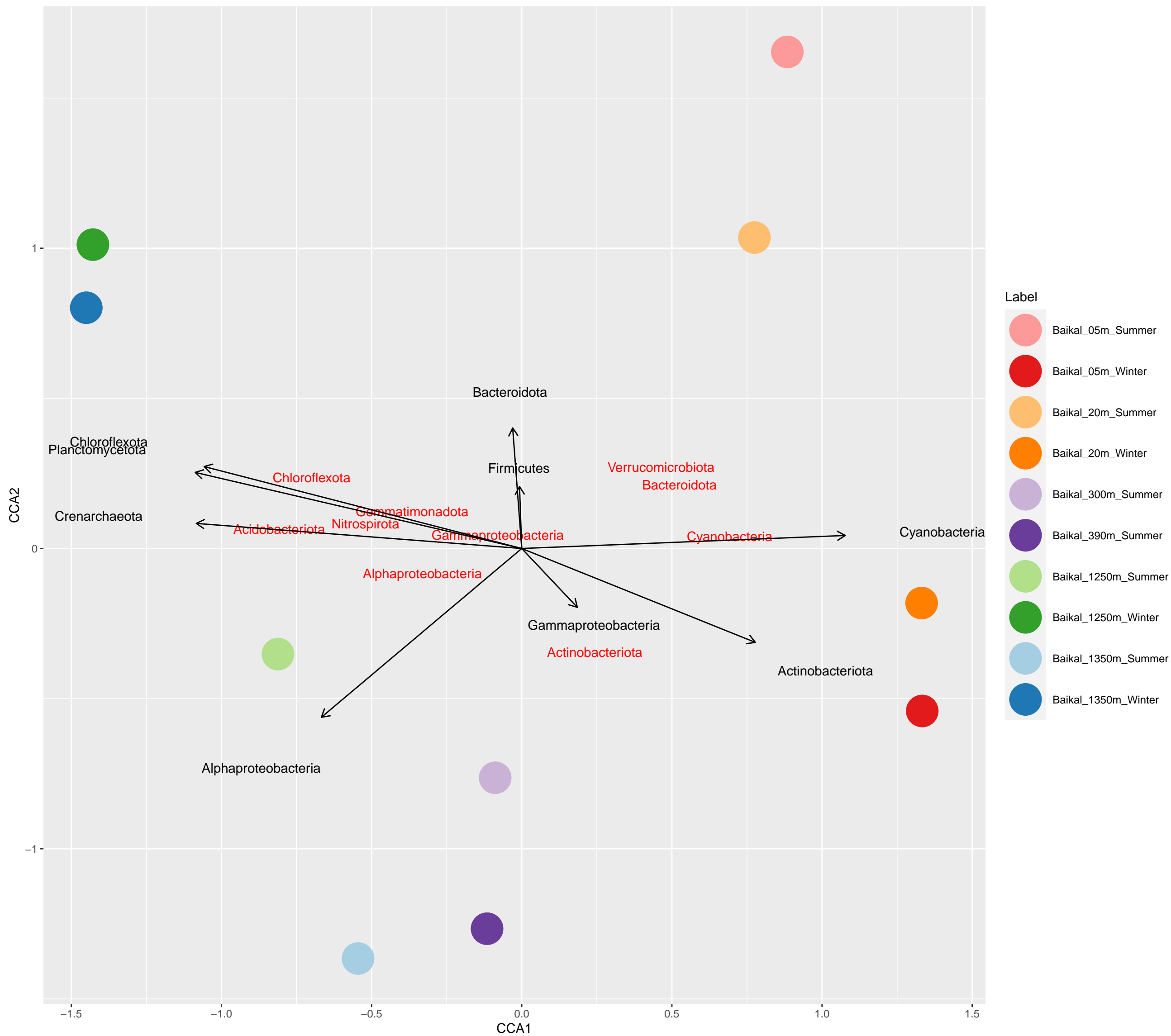

Supplement: Supplementary file 3 — Figure S1. Canonical Correspondence Analysis depicting the associations among the abundances of viruses (grouped at the level of phylum, or class in the case of Proteobacteria) and prokaryote taxa (grouped at the level of phyla, or class in the case of Proteobacteria) across samples. Coloured dots represent samples, red taxon names represent prokaryote abundances and arrows represent viral group abundances. (PDF 6.83 kb) [file 40168_2020_936_MOESM2_ESM.pdf]
